# Comparison of the efficacy of transcatheter arterial chemoembolization and sorafenib for advanced hepatocellular carcinoma

**DOI:** 10.3892/etm.2012.611

**Published:** 2012-06-15

**Authors:** HIROKI NISHIKAWA, YUKIO OSAKI, ERIKO IGUCHI, HARUHIKO TAKEDA, JUN NAKAJIMA, FUMIHIRO MATSUDA, AZUSA SAKAMOTO, SHINICHIRO HENMI, KEIICHI HATAMARU, SUMIO SAITO, AKIHIRO NASU, RYUICHI KITA, TORU KIMURA

**Affiliations:** Department of Gastroenterology and Hepatology, Osaka Red Cross Hospital, Tennoji-ku, Osaka 543-0027, Japan

**Keywords:** hepatocellular carcinoma, transcatheter arterial chemoembolization, sorafenib, survival, outcome

## Abstract

The aim of the present study was to compare overall survival between stage IVA or stage IVB hepatocellular carcinoma (HCC) patients who received transcatheter arterial chemoembolization (TACE) and those who were treated with sorafenib. This retrospective comparative study included 55 patients with stage IVA or IVB HCC in whom TACE was performed as an initial treatment (the TACE group) and 56 patients with stage IVA or IVB HCC to whom sorafenib was administered (the sorafenib group). We compared the overall survival between these two groups. In the TACE group, there were 46 stage IVA HCC patients and 9 stage IVB HCC patients. In the sorafenib group, there were 26 stage IVA HCC patients and 30 stage IVB HCC patients. Median overall survival times were 6.6 months in the TACE group and 9.2 months in the sorafenib group. The 1- and 2-year overall survival rates were 34.4 and 14.2%, respectively, in the TACE group and 34.0 and 6.7%, respectively, in the sorafenib group. In terms of overall survival, there was no significant difference between the two groups (P=0.814). In subgroup analyses, according to HCC stage [stage IVA (P=0.266) or stage IVB (P=0.183)] and Child-Pugh classification [Child-Pugh A (P=0.915) or Child-Pugh B (P=0.676)], there were also no significant differences between the two groups. In conclusion, our study results suggest that TACE could serve as a first-line treatment for stage IV HCC patients as well as sorafenib therapy.

## Introduction

Hepatocellular carcinoma (HCC) is a problem worldwide, particularly in Asian countries (1–3). Unlike most solid cancers, the incidence and mortality rates for HCC are projected to increase substantially in many countries over the next 20 years, mostly as a result of infections with hepatitis C and hepatitis B viruses (4). It has become possible to identify a group of patients with chronic liver disease who are at a high risk of developing HCC. In addition, improvements in diagnostic imaging have allowed early diagnosis of HCC. However, the majority of HCC patients are first seen when the disease has reached an advanced stage at which curative treatment is no longer possible (4).

Since HCC is considered to be chemoresistant in general, results of systemic chemotherapy have previously been disappointing (5). Sorafenib (Nexavar, Bayer Healthcare Pharmaceuticals), a multi-kinase inhibitor that blocks tumor growth and cell proliferation, was the first systemic chemotherapeutic agent found to improve the survival time of patients with advanced HCC in the SHARP and Asian Pacific trials (6,7). Sorafenib has opened a novel era for the treatment of advanced HCC. However, it is associated with a low tumor response rate, minimal survival advantage and high rates of adverse events (6,7).

Transcatheter arterial chemoembolization (TACE) is a procedure whereby an embolizing agent is injected into the hepatic artery to deprive the tumor of its major nutrient source via embolization of the nutrient artery, resulting in ischemic necrosis of the tumor with minimization of systemic side effects. It has become the most popular palliative treatment for patients with unresectable HCC (8–10). Patients with well preserved liver function and multi-nodular HCC without vascular invasion appear to be the best candidates for TACE (8–10). However, TACE is no longer considered to be contra-indicated in advanced HCC with portal vein tumor thrombus (PVTT) (11,12), and even in advanced HCC patients with extrahepatic metastasis, in cases in which extrahepatic spread is minimal and local control of liver tumors is considered more important, TACE is useful and may obtain survival benefits and has often been used in these cases in Japan (5,13). However, the long-term outcomes are less favorable in general for advanced HCCs treated with TACE, since the devascularization effect induced by TACE is transient, resulting in tumor progression (14).

Recently, concurrent or sequential treatment methods of advanced HCC with TACE and sorafenib with a manageable safety profile and a possibility of promising efficacy have been reported (15,16). However, regarding comparison of survival outcomes of advanced HCC patients treated with TACE and those treated with sorafenib, there have been no reliable data to the best of our knowledge to date.

The present study aimed to compare overall survival between stage IVA or IVB HCC patients who received TACE and those who were treated with sorafenib.

## Patients and methods

### 

#### Patients

This retrospective comparative study included 55 patients with stage IVA or IVB HCC in whom TACE was performed as an initial treatment (the TACE group) between April 2004 and November 2011 at the department of Gastroenterology and Hepatology, Osaka Red Cross Hospital, Japan and 56 patients with stage IVA or IVB HCC in whom sorafenib was administered (the sorafenib group) between June 2009 and October 2011 at our department. Since the aim of the present study was to compare clinical outcomes between stage IV HCC patients treated with TACE and those treated with sorafenib, 6 patients in whom TACE was performed as an initial treatment and thereafter sorafenib treatment was started were excluded in the present study. None of the TACE group patients received systemic chemotherapy and locoregional therapy other than TACE during the follow-up period. None of the sorafenib group patients received previous systemic chemotherapy. After patients were provided with sufficient information regarding TACE and sorafenib treatment, they themselves decided whether they were treated with TACE or sorafenib. Written informed consent was obtained from all patients prior to each treatment and this study protocol complied with all provisions of the Declaration of Helsinki.

#### Diagnosis of HCC

HCC was diagnosed using abdominal ultrasound and dynamic computed tomography (CT) scans (hyperattenuation during the arterial phase in all or some part of the tumor and hypoattenuation in the portal-venous phase), mainly based on the recommendations of the American Association for the Study of Liver Diseases (17). The presence of vascular invasion of the tumor was confirmed with the demonstration of a low-attenuation intraluminal mass expanding the portal vein, the bile duct, or the hepatic vein and/or filling defects in these vascular sites at dynamic CT. Arterial and portal phase dynamic CT images were obtained at approximately 30 and 120 sec, respectively, after injecting contrast material. Abdominal CT, chest CT, bone scintigraphy, brain CT and/or brain magnetic resonance imaging were performed prior to treatment in all stage IVB HCC patients. Diagnosis of stage IVB HCC was determined using these imaging modalities. Histopathological examination for metastasis was not performed. All eligible patients in the present study had bidimensionally measurable, inoperable HCC, no prior systemic treatments for HCC, an Eastern Cooperative Oncology Group (ECOG) performance status of 0 or 1, and a Child-Pugh classification of either A or B.

#### TACE procedure

TACE for HCC was performed in conformity with Japanese guidelines for this therapy, comprising catheterization via the femoral artery with super-selective cannulation to the hepatic artery feeding the target HCC (18). Farmorubicin (epirubicin hydrochloride; Pfizer, New York, NY, USA) was infused at 20–60 mg, mitomycin (mitomycin C; Kyowa Hakko, Tokyo, Japan) was infused at 4–14 mg, and Lipiodol (iodine addition products of ethyl esters of fatty acids obtained from poppy seed oil; Mitsui, Japan) was also injected at 2–15 ml according to the tumor size and tumor number. This was followed by embolization with gelatin (Spongel; Yamanouchi, Japan), which was injected slowly to prevent reflux into untreated segments. The sites of injection of the embolizing agents were segmental or subsegmental in all patients.

In the TACE group, after the initial TACE, another session of TACE was performed every 4–12 weeks until one of the following end points were reached: i) technical impossibility in performing TACE; ii) complete devascularization of the target HCC; iii) development of contraindications to TACE such as liver failure.

#### Sorafenib dose and treatment

Initiated sorafenib dose was determined considering factors such as patient’s body weight, performance status, and liver function. In all patients with Child-Pugh B, the initiated sorafenib dose was 200 mg twice a day (b.i.d.). Sorafenib treatment continued until one of the following criteria was met: disease progression, unacceptable drug-related toxicities or patient’s wish for discontinuation.

#### Evaluation of treatment efficacy

Tumor response was assessed at 8–12 weeks according to the modified Response Evaluation Criteria in Solid Tumors (RECIST) criteria using dynamic CT scans. The change in viable perfused tumor volume of the targeted lesions as measured on the arterial phase imaging before and after treatment was evaluated (19).

#### Follow-up

In the TACE group, follow-up consisted of monthly blood tests and monitoring of tumor markers. Dynamic CT scans were obtained every 8–12 weeks during the follow-up period. No patients were lost to follow-up in the TACE group. In the sorafenib group, follow-up consisted of weekly blood tests for the purpose of detecting adverse events and monitoring of tumor markers. Dynamic CT scans were obtained every 8–12 weeks during the follow-up period. No patients were lost to follow-up in the sorafenib group.

#### Statistical analysis

The primary end point was overall survival. It was calculated from the date of first diagnosis with stage IVA or stage IVB HCC using imaging modalities until death from any cause or the last follow-up. Differences between the two groups were analyzed using the unpaired t-test for continuous variables, and the categorical variables were analyzed using the Fisher’s exact test. The overall survival curves were generated using the Kaplan-Meier method and compared using the log-rank test. All statistical tests were two-sided. All data were analyzed using SPSS software, version 9.0 (SPSS Inc., Chicago, IL, USA) for Microsoft Windows. Data are expressed as means ± standard deviation (SD). Values of P<0.05 were considered to indicate statistical significance.

## Results

### Baseline characteristics

Baseline characteristics between the TACE group and the sorafenib group are shown in Table I. There were 55 patients in the TACE group and 56 in the sorafenib group. In the TACE group, there were 46 stage IVA HCC patients and 9 stage IVB HCC patients, respectively. In the sorafenib group, there were 26 stage IVA HCC patients and 30 stage IVB HCC patients, respectively. Fifty-one patients (91.1%) in the sorafenib group had received previous locoregional therapies such as percutaneous thermal ablation, percutaneous ethanol injection therapy or transcatheter arterial infusion chemotherapy without embolization, and all patients in the sorafenib group received at least one dose of sorafenib. In terms of HCC stage, there was a significant difference between the two groups (P<0.001). However, in terms of gender, age, etiology of liver disease, maximum tumor size, Child-Pugh classification, and laboratory data including tumor markers and body mass index, there were no significant differences between these two groups.

### Overall survival

**Figure 1:**
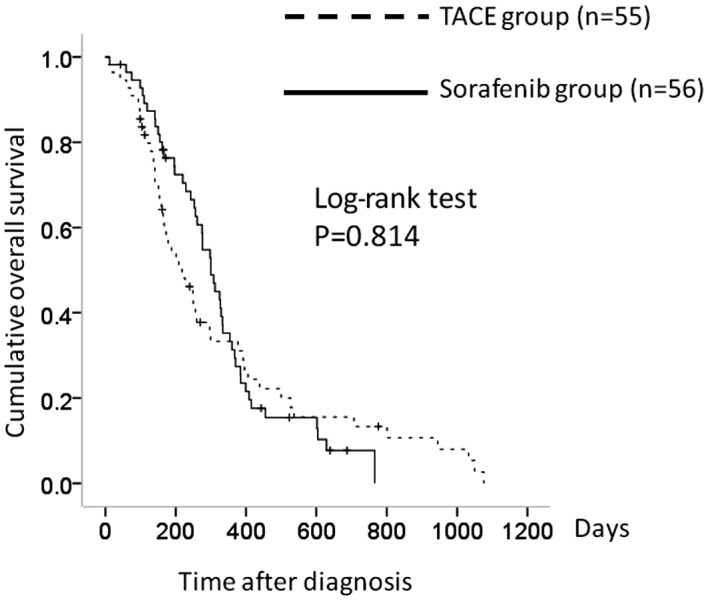
Cumulative overall survival between the transcatheter arterial chemoembolization (TACE) group and the sorafenib group. The 1- and 2-year overall survival rates were 34.4 and 14.2%, respectively, in the TACE group and 34.0 and 6.7%, respectively, in the sorafenib group. In terms of overall survival, there was no significant difference between the two groups (P=0.814).

Median overall survival times were 6.6 months in the TACE group and 9.2 months in the sorafenib group. The 1- and 2-year overall survival rates were 34.4 and 14.2%, respectively, in the TACE group and 34.0 and 6.7%, respectively, in the sorafenib group. In terms of overall survival, there was no significant difference between the two groups (P= 0.814) (Fig. 1).

### Subgroup analyses

#### Comparison between the TACE and sorafenib group patients with stage IVA HCC

**Figure 2:**
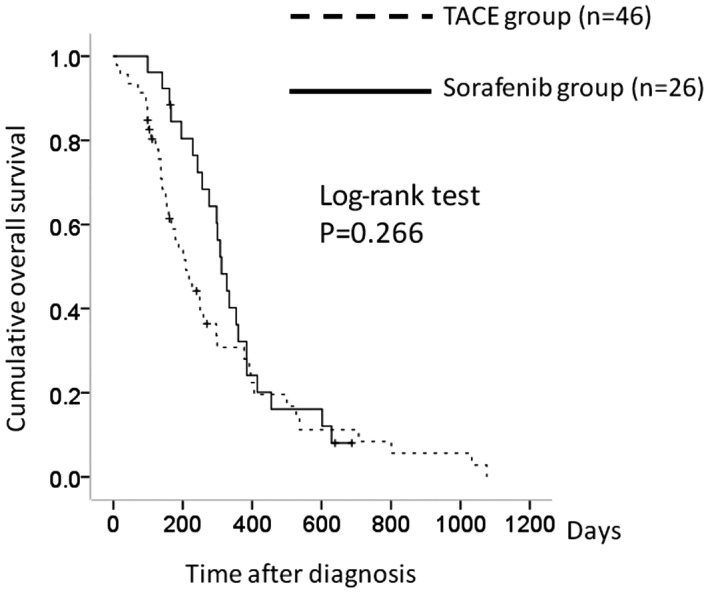
Cumulative overall survival between the transcatheter arterial chemoembolization (TACE) group patients with stage IVA hepatocellular carcinoma (HCC) (n=46) and the sorafenib group patients with stage IVA HCC (n=26). The 1-year overall survival rates were 30.6% in the TACE group and 32.8% in the sorafenib group, respectively. In terms of overall survival, there was no significant difference between the two groups (P=0.266).

There were 46 patients in the TACE group with stage IVA HCC and 26 in the sorafenib group. The 1-year overall survival rates were 30.6% in the TACE group and 32.8% in the sorafenib group. In terms of overall survival, there was no significant difference between the two groups (P=0.266) (Fig. 2).

#### Comparison between the TACE and sorafenib group patients with stage IVB HCC

**Figure 3:**
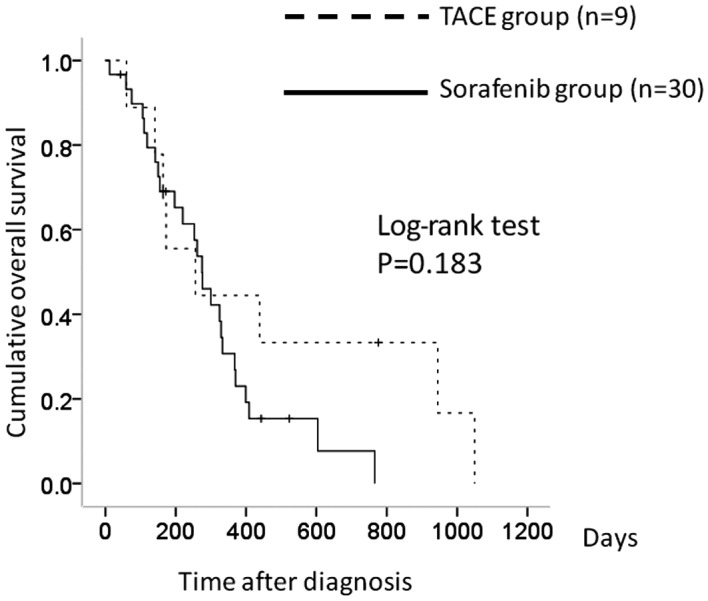
Cumulative overall survival between the transcatheter arterial chemoembolization (TACE) group patients with stage IVB hepatocellular carcinoma (HCC) (n=9) and the sorafenib group patients with stage IVA HCC (n=30). The 1-year overall survival rates were 43.5% in the TACE group and 30.2% in the sorafenib group. In terms of overall survival, there was no significant difference between the two groups (P=0.183).

There were 9 patients in the TACE group with stage IVB HCC and 30 in the sorafenib group. The 1-year overall survival rates were 43.5% in the TACE group and 30.2% in the sorafenib group. In terms of overall survival, there was no significant difference between the two groups (P=0.183) (Fig. 3).

#### Comparison between the TACE and sorafenib group patients with Child-Pugh A

**Figure 4:**
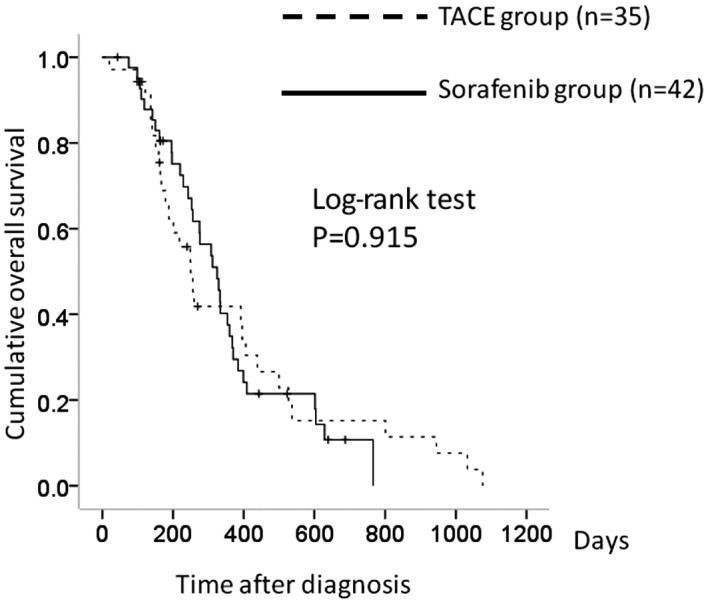
Cumulative overall survival between the transcatheter arterial chemo embolization (TACE) group patients with Child-Pugh A (n=35) and the sorafenib group patients with Child-Pugh A (n=42). The 1-year overall survival rates were 41.1% in the TACE group and 30.4% in the sorafenib group. In terms of overall survival, there was no significant difference between the two groups (P=0.915).

There were 35 patients in the TACE group with Child-Pugh A and 42 in the sorafenib group. The 1-year overall survival rates were 41.1% in the TACE group and 30.4% in the sorafenib group. In terms of overall survival, there was no significant difference between the two groups (P=0.915) (Fig. 4).

#### Comparison between the TACE and sorafenib group patients with Child-Pugh B

**Figure 5:**
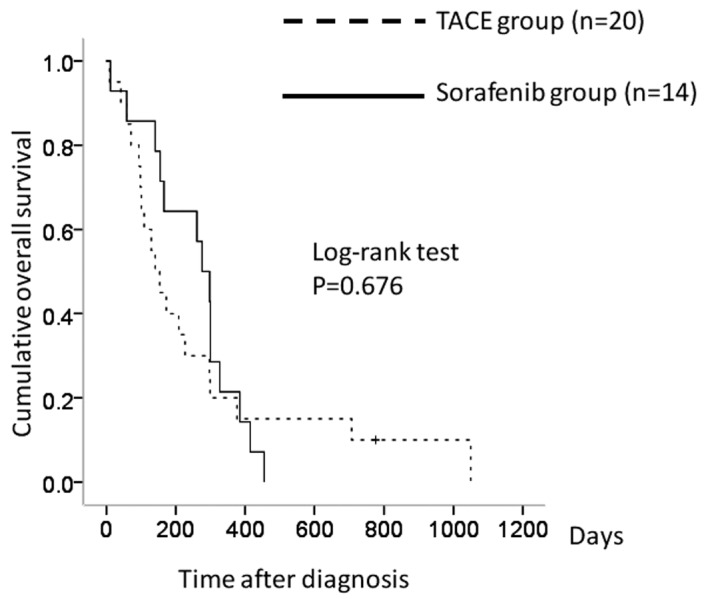
Cumulative overall survival between the transcatheter arterial chemoembolization (TACE) group patients with Child-Pugh B (n=20) and the sorafenib group patients with Child-Pugh A (n=14). The 1-year overall survival rates were 20.0% in the TACE group and 21.4% in the sorafenib group, respectively. In terms of overall survival, there was no significant difference between the two groups (P=0.676).

There were 20 patients in the TACE group with Child-Pugh B and 14 in the sorafenib group. The 1-year overall survival rates were 20.0% in the TACE group and 21.4% in the sorafenib group. In terms of overall survival, there was no significant difference between the two groups (P=0.676) (Fig. 5).

#### Outcomes in the TACE group

During the follow-up period, a mean of 3.0 (range, 1–9) sessions of TACE were performed in the TACE group. Eighteen patients (32.7%) received 1 session and 37 (67.3%) received more than 1 session of TACE. Partial response (PR) was obtained in 10 patients (18.2%). Stable disease (SD) was observed in 31 patients (56.4%). Progressive disease (PD) was observed in 14 patients (25.5%). The objective response and disease control rates in the TACE group were 18.2 and 74.5%, respectively.

#### Adverse events related to TACE

The majority of patients suffered self-limited post-embolization syndrome consisting of low-grade fever, appetite loss, abdominal pain, nausea or mild vomiting, which were effectively controlled and improved within a few days. During the follow-up period, 23 clinical adverse events with grade 3 or higher were observed in the TACE group as determined with National Cancer Institute Common Terminology Criteria for Adverse Events, version 3.0 (20). The details were as follows: appetite loss in 7 patients (12.7%), hepatotoxicity in 6 patients (10.9%), general fatigue in 7 patients (12.7%) and high grade fever in 3 patients (5.5%), respectively. All improved during hospitalization and no patients died of TACE-related adverse events.

#### Outcomes in the sorafenib group

In the sorafenib group, the median interval between first diagnosis date of stage IVA or IVB HCC and initiation date of sorafenib treatment was 40 days (range, 1–203 days). Median duration of sorafenib therapy was 73 days (range, 4–377 days) for all patients treated with sorafenib. In 42 patients with Child-Pugh A, median duration of sorafenib therapy was 82 days (range, 4–377 days). In 14 patients with Child-Pugh B, median duration of sorafenib therapy was 35 days (range, 10–287 days). In 16 patients (28.6%), sorafenib 400 mg b.i.d. was started. In 40 patients (71.4%), sorafenib 200 mg b.i.d. was started. In 16 of 16 patients (100%) with initiated sorafenib 400 mg b.i.d., dose reductions were required. In 29 of 40 patients (72.5%) with initiated sorafenib 200 mg b.i.d., dose reductions were required. Complete response was obtained in 1 patient (1.8%). PR was obtained in 5 patients (8.9%). SD was observed in 22 patients (39.3%). PD was observed in 26 patients (46.4%). In 2 patients (3.6%), treatment efficacy was not determined, since evaluation using dynamic CT was not performed. The objective response and disease control rates in the sorafenib group were 11.1 and 51.9%, respectively.

#### Adverse events associated with sorafenib treatment

During the follow-up period, 38 clinical adverse events with grade 3 or higher were observed in the sorafenib group as determined with National Cancer Institute Common Terminology Criteria for Adverse Events, version 3.0 (20). The details were as follows: rash in 2 patients (3.6%), hand-foot syndrome in 4 patients (7.1%), diarrhea in 8 patients (14.3%), appetite loss in 4 patients (7.1%), hepatotoxicity in 10 patients (17.9%), general fatigue in 5 patients (8.9%), high-grade fever in 3 patients (5.4%), and lung toxicity in 2 patients (3.6%).

#### Causes of discontinuation of sorafenib

In the sorafenib group, 45 patients (80.4%) discontinued sorafenib treatment. Causes of discontinuation were as follows: tumor progression in 21 patients, serious adverse events in 23 patients and patient’s wish in 1 patient.

#### Causes of death

During the follow-up period, 48 patients (87.3%) died in the TACE group. Mortality in the TACE group was due to tumor progression in 34 patients (61.8%), liver failure in 13 patients (23.6%) and pneumonia in 1 patient (1.8%). During the follow-up period, 48 patients (85.7%) died in the sorafenib group. Mortality in the sorafenib group was due to tumor progression in 35 patients (62.5%), liver failure in 8 patients (14.3%) and pneumonia in 5 patients (8.9%).

## Discussion

To our best knowledge, there have been no reliable data to date with regard to comparison between conventional TACE and sorafenib treatment for advanced HCC with vascular invasion and/or extrahepatic metastasis. Therefore, in the present study, we aimed to compare overall survival between stage IV HCC patients treated with conventional TACE and those treated with sorafenib.

Sorafenib was the first systemic chemotherapeutic agent to demonstrate a significant improvement in overall survival in patients with advanced HCC (6,7). However, Niu *et al* reported in their prospective comparative study that TACE was an effective treatment method for advanced HCC with PVTT compared to conservative treatment (21). Luo *et al* also reported in their prospective study that TACE was safe and feasible in selected HCC patients with PVTT and that it had survival benefit over conservative treatment (22). Chung *et al* also reported in their large retrospective study that TACE for advanced HCC patients with main portal vein invasion can be performed safely and may improve overall survival (23). Thus, several studies with favorable outcome in patients with stage IV HCC who received TACE have been reported. In the present study, in terms of overall survival, there were no significant differences between the TACE and the sorafenib groups. Our study results suggest that TACE could be a first-line treatment for stage IV HCC.

In terms of the objective response rate, there was no significant difference between the two groups (TACE group, 18.2%; sorafenib group, 11.1%; P=0.418). This result also suggests that TACE can be considered as a therapeutic option for the treatment of stage IV HCC.

In terms of the disease control rate, there was a significant difference between the two groups (TACE group, 74.5%; sorafenib group, 51.9%; P=0.017). Although the reason for this is unclear, TACE may be more effective at suppressing disease progression in stage IV HCC than sorafenib therapy.

Serum vascular endothelial growth factor (VEGF) levels increase with advancing HCC stages (24). Treatment of HCC with TACE is known to induce VEGF expression (25,26). In particular, in patients with incomplete response to TACE, TACE can induce the up-regulation of VEGF (24). Serum VEGF level was an independent predictor of survival in patients with advanced HCC (27,28). In the present study, there were 45 patients (81.8%) who did not obtain CR or PR in the TACE group. In these patients, in order to suppress VEGF and malignant angiogenesis, concurrent or sequential therapy with molecular targeted drugs such as sorafenib may be effective to optimize outcome (29).

Currently, in Japan, advanced HCCs are treated by hepatologists or radiologists. The former may be less familiar with the side effects of anticancer drugs, and the latter may not be prepared to manage problems related to underlying liver cirrhosis. Collaboration between hepatologists and radiologists is therefore essential to optimize outcome in the treatment of advanced HCC patients.

There are several limitations in the present study. First, this was a retrospective study. Second, in the sorafenib group, in 40 patients (71.4%), sorafenib 200 mg b.i.d. was started, leading to underestimated outcomes of patients treated with sorafenib, although in the SHARP and Asian Pacific trials, sorafenib 400 mg b.i.d. was started in all eligible patients. (6,7). Third, in the TACE group, previous therapies for HCC were not performed, whereas in the sorafenib group, previous locoregional therapies were performed, leading to bias. Therefore, a large prospective study will be required in the future. However, our study results demonstrated that in terms of overall survival, including subgroup analyses, there were no significant differences between the TACE group and the sorafenib group. In conclusion, TACE for stage IV HCC can be a first-line treatment as well as sorafenib therapy.

## Figures and Tables

**Table I:** Baseline characteristics between the TACE group and the sorafenib group.

	TACE group (n=55)	Sorafenib group (n=56)	P-value
Gender (M/F)	42/13	46/10	0.490^a^
Age (years)	67.9±10.0	69.1±12.0	0.563^b^
Etiology of liver disease			
B/C/B,C/non-B, non-C	7/29/4/15	9/29/1/17	0.575^a^
Child-Pugh classification			
Child-Pugh A/Child-Pugh B	35/20	42/14	0.221^a^
HCC stage			
Stage IVA/stage IVB	46/9	26/30	<0.001^a^
Maximum tumor size (cm)	7.6±3.1	6.3±4.4	0.087^b^
Total-bilirubin (mg/dl)	1.03±0.69	0.91±0.50	0.311^b^
Serum albumin (g/dl)	3.51±0.56	3.66±0.49	0.120^b^
Platelets (×10^4^/mm^3^)	16.1±7.7	15.3±8.2	0.595^b^
ALT (IU/l)	66.5±86.8	45.7±39.7	0.106^b^
Prothrombin time (%)	85.8±17.0	82.7±12.4	0.268^b^
AFP (ng/ml)	25,223.4±96,684.3	17,945.7±92,746.3	0.674^b^
PIVKAII (mAU/ml)	50,535.4±83,206.0	28,613.6±117,254.6	0.259^b^
Body mass index (kg/m^2^)	22.4±3.3	22.2±4.1	0.756^b^

Data are expressed as the number of patients or the mean ± standard deviation. TACE, transcatheter arterial chemoembolization; HCC, hepatocellular carcinoma; ALT, alanine aminotransferase; AFP, α-fetoprotein; PIVKAII, protein-induced vitamin K absence or antagonist II.

a Fisher’s exact test;

b Student’s t-test. B, hepatitis B virus; C, hepatitis C virus.
